# p53-Autophagy-Metastasis Link

**DOI:** 10.3390/cancers10050148

**Published:** 2018-05-18

**Authors:** Tatiana V. Denisenko, Anastasia D. Pivnyuk, Boris Zhivotovsky

**Affiliations:** 1 Faculty of Medicine, M.V. Lomonosov Moscow State University, 119991 Moscow, Russia; de_tanya@yahoo.com (T.V.D.); a.pivnuk@gmail.com (A.D.P.); 2 Institute of Environmental Medicine, Division of Toxicology, Karolinska Institutet, Box 210, SE-171 77 Stockholm, Sweden

**Keywords:** autophagy, apoptosis, p53, cancer, metastasis

## Abstract

The tumor suppressor p53 as the “guardian of the genome” plays an essential role in numerous signaling pathways that control the cell cycle, cell death and in maintaining the integrity of the human genome. p53, depending on the intracellular localization, contributes to the regulation of various cell death pathways, including apoptosis, autophagy and necroptosis. Accumulated evidence suggests that this function of p53 is closely involved in the process of cancer development. Here, present knowledge concerning a p53-autophagy-metastasis link, as well as therapeutic approaches that influence this link, are discussed.

## 1. Introduction

Being one of the major suppressors of tumorigenesis, p53 is involved in regulation of the cell cycle, cell death, maintenance of genome integrity and DNA repair. Therefore, it is traditionally considered a “guardian of the genome.” In recent years, however, p53 has also been reported to possess some novel “non-canonical” functions, including the regulation of metabolism and autophagy.

The structure of p53 reflects the complexity of its functions. Based on gel electrophoresis analysis, it is a nuclear protein with a molecular mass of 53 kD; however, the real mass of this protein is 43.7 kDa. It contains 393 amino acids and acts mainly as a transcription factor for multiple genes [1,2,3]. It has a modular domain structure and is active as a tetramer, while each of the five discrete p53 domains are well defined and correspond to a specific function. The N-terminal transactivation domain (TAD) is critical for binding to the components of the transcription initiation complex and variety of regulatory proteins, which modulate p53 activity via posttranslational modifications [4,5]. The TAD is followed by a proline-rich region, which is considered to play a role in the regulation of p53 stability [6]. The central (core) DNA-binding domain sequence specifically binds to double-stranded target DNA with different degrees of affinity. It is high for genes involved in regulation of the cell cycle, and lower for genes that control apoptosis [7,8]. The tetramerization domain permits the oligomerization of p53, while the C-terminal negative autoregulatory domain is thought to be a negative regulator of sequence-specific DNA-binding, but its exact role is controversial [9]. The C-terminal and N-terminal regions are intrinsically disordered regions, which means that they lack well-structured three-dimensional folds in their native condition, being conformationally flexible and thermodynamically unstable, but upon binding to various proteins, they obtain a well-defined full amphipathic α-helix [10,11,12]. Intrinsic disorder is distinctive for proteins that are involved in multiple protein-protein interactions and reflects the versatility of their functions. The intracellular concentration and activity of p53 are accurately controlled and finely regulated on different levels. The expression of the *p53* gene is modified by a number of transcription activators (i.e., C/EBPβ and RBP-Jκ) [13] and repressors (i.e., CTCF), miRNAs and WRAP53 [14] an antisense RNA, which is necessary for p53 induction upon DNA damage. The protein also undergoes various posttranslational modifications, which notably affect its activity, and the most prominent negative regulator of p53 is the MDM2 (Mouse double minute 2 homolog) protein [15,16]. It serves as an E3 ubiquitin ligase and catalyzes p53 polyubiquitination for subsequent proteasome degradation. In response to various stimuli p53 is stabilized; for example, DNA-damage leads to the phosphorylation of the MDM2-binding site of p53, thereby, blocking MDM2-mediated degradation. Other posttranslational modifications of p53 include phosphorylation by stress-activated kinases, including ATM (ataxia telangiectasia mutated), CK1 (casein kinase 1) and AMPK (AMP-activated protein kinase) [17] acetylation by its transcriptional coactivator p300/CBP [18] and conformational changes, which are induced by Pin1 [19].

Structural peculiarities of p53 give a clue toward understanding the diversity of its functions. First classified as an oncogene, p53 exhibits various antiproliferative functions, and suppresses growth and tumorigenesis in cell culture. The best-understood, canonical functions of p53 include regulation of the cell cycle and cell death. It may induce cell cycle arrest at G1/S and G2/M checkpoints in response to various disturbances through transactivation of p21 and 14-3-3σ, respectively [20,21]. Under certain circumstances, p53 may promote a permanent cell cycle arrest called senescence via interaction with the retinoblastoma (Rb) gene product [22] The role of p53 in the regulation of different types of cell death is the most extensively studied. Upon irreparable DNA damage p53 transactivates numerous proapoptotic genes, including PUMA, Noxa, Bax and other pro-apoptotic members of the Bcl-2 family and thus promotes apoptosis [23]. In addition, it has also been shown that p53 via activation of Bax is able to induce mitochondrial outer membrane permeabilization (MOMP), which results in the release of cytochrome c and other pro-apoptotic proteins from the intermembrane space to cytosol and induction of apoptosis [24]. Moreover, recent studies have implicated p53 in the regulation of autophagy via the AMPK/TSC2-mTOR signaling pathway. Importantly, two more p53-inducible signaling molecules, DRAM (damage-regulated autophagy modulator) and p14ARF, are involved in the regulation of the autophagy pathway and are also able to activate apoptosis. Thus, p53 is thought to indirectly facilitate further progression of apoptosis due to the activation of autophagy, especially in cancer cells [25,26] p53 has also been found to repress autophagy to different degrees, depending on the phase of the cell cycle [27]. Finally, p53 was also shown to increase genomic stability via suppression of retrotransposons, whose overexpression leads to mutagenesis [28]. Hence, p53 ultimately reduces the risk of tumorigenesis, as it promotes either the repair or elimination of cells with damaged DNA. In recent years data have also been obtained about non-canonical functions of p53, especially its impact on metabolism of tumor cells, including glycolysis and oxidative phosphorylation, but their contribution to the tumor suppression activity of p53 still remains speculative and requires further investigation [29].

p63 and p73 are close homologues of p53 and they are all members of the p53 family of transcription factors. They share a similar domain structure, but p53 lacks a specific C-terminal region, which is alternatively spliced in p63 and p73. Thus, the latter exist as different isoforms, which exhibit different, even opposite functions. Full-length isoforms (TAp63 and TAp73) act synergistically with p53. They can bind to classical DNA p53-binding sites and consequently induce cell cycle arrest, apoptosis and senescence in the same manner as p53 [30]. Amino-terminally truncated ΔN isoforms of p73 are transcriptionally inactive as they lack a N-terminal TAD and behave as competitive inhibitors of the full-length forms. p63 and p73 are rarely mutated in cancers, in contrast to p53 which is mutated in roughly 50% of cases, and, therefore, can partly assume functions of p53 [31].

Mutations of p53 lead to increased susceptibility to tumor transformation and are found in over 50% of cancers. They are considered to be necessary for the development of most tumors, especially ovarian cell carcinoma, squamous lung cancer and triple-negative breast cancer. Their presence often contributes to rapid disease progression and resistance to conventional therapeutics and is associated with poor prognosis [32]. These mutations can be divided into two groups: loss-of-function and gain-of-function, the latter of which underlies novel properties of mutant p53. They usually affect the ability of p53 to bind DNA specifically or to interact with other proteins, including various transcription factors [33] and proteins, which are not directly involved in the regulation of gene expression. Many p53 mutants obtain the ability to promote tumorigenesis through augmented proliferation, invasion, motility and cell survival [34,35]. Moreover, mutant p53 can induce genomic instability, chemoresistance and proliferation in different fashion. There is a growing body of evidence that mutations of p53 also play a role in cell reprogramming, expansion and interaction with tumor stroma, especially with cancer-associated fibroblasts (CAFs), which secrete various cytokines and growth factors that mediate mutant p53-dependent invasion and metastasis [36,37,38]. Moreover, inactivation of wt (wild type) p53 augments inflammation-induced tumorigenesis, partly due to reciprocal NFκB overactivation [39] but also because it promotes production of multiple proinflammatory cytokines by macrophages [40] enhances IL-6 production and, therefore, differentiation of Th cells, and compromises Treg differentiation [41]. In respect to autophagy, mutant p53 has been shown to stimulate mTOR and AMPK and thus suppress autophagy in cancer cells [42].

## 2. Autophagy: General Aspects

Macroautophagy (hereafter referred to as autophagy) is a highly conserved catabolic process that captures and degrades misfolded proteins and cellular organelles and sustains cell survival during different stress processes [43,44]. Autophagy begins with the formation of double-membrane structures, known as “autophagosomes”, that engulfing cellular cytoplasmic constituents and subsequently fuse with lysosomes to form an autophagolysosome structure to degrade their contents [43]. Autophagy plays an important role in maintaining cellular homeostasis and is therefore constitutively active at a basal level in most cell types. In physiological circumstances, basal levels of autophagy are normally low; however, under different stress conditions, such as those induced by nutrient starvation, organelle damage, accumulation of abnormal proteins, or during development and cell differentiation, autophagy is additionally enhanced to meet the cellular needs [45,46].

Unlike their normal counterparts, cancer cells may have high levels of basal autophagy and can be dependent on autophagy for survival. It is now well known that autophagy may play a tumor-suppressing or tumor-promoting roles depending on the context [44,47,48]. Thus, at early stages of carcinogenesis autophagy has been shown to suppress tumor formation by removing damaged organelles/proteins and limiting cell proliferation and genomic instability [49]. Moreover, genes that negatively regulate the mammalian target of rapamycin (mTOR), the main regulator of autophagy, such as *AMPK, LKB1, PTEN* and *TSC1/2* induce autophagy while, conversely, oncogenes that promote mTOR signaling, such as class I *PI3K*, *AKT, Ras* and RHEB inhibit autophagy [50,51]. In line with this view, monoallelic loss of the autophagy-related gene *Beclin 1* has been detected in 40% to 75% of human ovarian, prostate, and breast cancers. *Beclin 1*-deficient mice were shown to be tumor prone [52]. In contrast, increased stimulation of autophagy provided by Beclin 1 overexpression can block tumor development [44,45]. These observations confirm that *Beclin 1* may act as a tumor suppressor gene involved in the pathogenesis of human cancers and that autophagy may prevent the development of these tumors [53,54].

Heterozygous deletion of several other core autophagy genes is reported to promote a tumor-suppressing role of autophagy in cancer [45]. Thus, deficiency of the UV radiation resistance-associated gene (UVRAG), a positive Beclin 1/PI(3)K complex regulator, leads to the autophagy inhibition contributing to the development of human colon and gastric cancers [55,56]. Moreover, Bax-interacting factor-1 (Bif-1), which forms a multiprotein complex with UVRAG and Beclin 1, has been found to be lost in gastric and prostate cancers [57]. Activation of the ATG13–ULK–FIP200 complex is required for activation of phagophore formation and also involved in regulation of nutrient starvation-induced autophagy [58,59]. Since the molecular machinery of the autophagosome formation includes the TORC1-ULK1-VPS34-Beclin 1 complex, nutrient deprivation and energy crisis identified in many tumors may lead to the inhibition of the mTORC1 pathway and activation of ULK1 kinase activity [60,61]. This activation results in phosphorylation of Beclin 1 on Ser14 and initiates the proautophagy VPS34 complexes to promote autophagy. Decreased ULK1 expression has been shown to be associated with cancer progression, suggesting ULK1 as a novel prognostic biomarker for breast cancer [62].

As well as mitochondria being considered to be the main source of ROS (Reactive Oxygen Species), autophagy can carry out its tumor suppressor function by enabling the elimination of damaged mitochondria, thereby preventing ROS accumulation [63]. This type of selective autophagy, called “mitophagy”, is regulated by several molecular pathways, including NIX/BNIP3L and PARKIN (PARK2)/PTEN-induced putative kinase1 (PINK1) [64,65] Nix/BNIP3L interacts with GABA RAP and GABA RAPL1 at the autophagosome and targets mitochondria for degradation [66] PARKIN/PINK1 allows the selective degradation of damaged and dysfunctional mitochondria in response to mitochondrial membrane depolarization induced by ROS [67]. It has been previously shown that elimination of damaged mitochondria by autophagy leads to decreased ROS production, thereby limiting the tumor-promoting effect of ROS [68]. Consequently, autophagy inhibition, following *ATG5* or *ATG7* deletion, leads to chronic oxidative stress, accumulation of damaged mitochondria, tissue damage and inflammation which all favor tumor initiation [69,70].

Defects in autophagy are associated with the accumulation of aggregated proteins and the autophagy substrate SQSTM1/p62, a ubiquitin-binding protein that is a target of cargo-selective autophagy. Such events are linked to the increased production of ROS, ER (endoplasmic reticulum) stress and activation of the DNA damage response [51]. Knockdown of SQSTM1/p62 in autophagy-defective cells prevents ROS accumulation and the DNA damage response [49]. The connection between unappropriated autophagy and SQSTM1/p62 accumulation and tumor formation was also detected when *SQSTM1/p62^-/-^* mice were protected from Ras-induced lung carcinomas compared with wt animals [71].

Finally, autophagy also represents the mechanism that may limit tumorigenesis by restraining tumor necrosis and chronic inflammation, which are mediated by the release of proinflammatory high-mobility group box protein 1 (HMGB1) [72]. This protein secreted by necrotic and immune cells binds different membrane receptors such as macrophage-1 antigen (Mac-1) or toll-like receptors (TLR2, 4 and 9) promoting proinflammatory response dependent on cytokines and leads to tumor progression [63,73]. The interplay between inflammatory cytokines and cancer remains unclear given the pro- and anti-tumor effect of these molecules depending on the tumor context.

While autophagy has a tumor-suppressing role in the early stage of carcinogenesis, in advanced cancers it often acts as a tumor survival or even tumor promoter mechanism [45]. This is mostly due to the fact that the autophagy level is significantly higher in the central part of the solid than on the periphery [47]. This suggests that autophagy in some tumors also acts as an adaptive mechanism enabling their advancement in the absence of key survival factors.

A high level of autophagy is detected in cancer cells bearing K-ras mutation promoting their proliferation and survival, whereas the deletion of core autophagy genes in tumor cells has been shown to stimulate the induction of cell death [48,74]. Thus, *ATG7* deletion in a K-ras-driven non-small cell lung cancer (NSCLC) significantly reduces tumor growth. Moreover, the ablation of ATG7 leads to the progression of adenomas and adenocarcinomas harboring K-ras mutation to benign oncocytomas, tumors characterized by the accumulation of dysfunctional mitochondria. ATG7 deficiency activates p53, which in turn contributes to tumor inhibition, as co-deletion of p53 in part rescue impairment of tumor growth [75]. Likewise, deletion of p53 impedes tumor progression promoted by autophagy in a mouse model of pancreatic cancer [76].

The role of autophagy in cancer is highly dependent on the type of tumor and its developmental stage. Activation or inactivation of autophagy can contribute differently to tumorigenesis. Reduced autophagy can contribute to tumor progression, whereas increased autophagy may be a mechanism for tumor survival under hypoxic, metabolic or therapeutic stress conditions. Thus, the modulation of the autophagy process is a promising, but complex, therapeutic strategy for the enhancement of anticancer treatments.

## 3. Cross-Talk between p53 and Autophagy is Essential for Cancer Development

Recent evidence suggests that p53 may also contribute to the relationship between autophagy and malignancy [77,78]. As mentioned above, p53 induces different cellular responses including the cell cycle arrest, senescence and apoptosis modulating the expression of target genes [5] p53 has also been shown to play an important role in autophagy regulation which depends on its location in different intracellular compartments [79]. Thus, nuclear p53 acts as a pro-autophagic factor whereas, in the cytoplasm, p53 inhibits the induction of autophagy.

In the nucleus, p53 induces autophagy through regulation of the mTOR pathway in a transcription-dependent manner. Many p53 target genes, including PTEN, TSC2 and AMPKβ, are known to negatively regulate mTOR [80]. The crucial link between p53 and mTORC1 activity is identified in the form of two p53 target genes namely Sestrin1 and Sestrin2 [81] p53 induces Sestrin in a response to DNA damage and oxidative stress. Sestrin stimulates AMPK-mediated TSC activation leading to the inhibition of mTORC1. In response to stresses, induced by nutrient starvation or rapamycin, Sestrin2 helps with the induction of autophagy [81].

Another link between p53and autophagy could be provided by p14ARF signaling. The ARF protein, encoded by *CDKN2A* gene, is considered to be a tumor suppressor, and has been shown to regulate p53 positively through inhibition of MDM2-induced degradation [82,83]. At the same time, full-length ARF has been shown to induce autophagy, whereas a short isoform of ARF (smARF) has been suggested to promote mitophagy. Furthermore, the mutation in *CDKN2A* exon2, suppresses the ability of ARF to induce autophagy [84].

p53 has also been shown to induce the autophagy pathway through DRAM, which is a lysosomal protein that modulates autophagy in response to nutrient starvation [85,86,87]. It can regulate autophagosomes accumulation and generate autolysosomes stimul, ating autophagosome-lysosome fusion. Another p53-target gene, ISG20L1 or AEN, was recently identified as a modulator of autophagy. Interestingly, ISG20L1 could be regulated by all three p53 family members (p53, p63 and p73) at transcriptional level, whereas deletion of ISG20L1 led to a decreased level of autophagy and associated genotoxic stress. Moreover, several other autophagy-associated genes have been shown to be regulated by all p53-family members. Indeed, p73 can promote autophagy binding to the promoters of ATG5, ATG7 and UVRAG [88] p63 has also been shown to interact with several genes associated with autophagy including ULK1, ATG5 and ATG7, as well as indirectly regulating autophagy through the transcription of miRNAs [89]. Thus, p53 family members may substitute for each other in autophagy regulation. However, their functions do not completely overlap. As mentioned above, p73 could be inhibited by mTOR, whereas p53 was regulated positively by mTOR [79]. Similarly to p53, p73 was shown to activate DRAM; however, its activation was not required for autophagy regulation by p73 [90]. These results additionally strengthen the link between autophagy and p53 signaling [91].

Meanwhile, inactivation of cytoplasmic p53 might trigger autophagy, suggesting that the nonnuclear p53 pool is a potent autophagy repressor [79,92]. Indeed, it was demonstrated that cytoplasmic p53 inhibits autophagy suppressing AMPK and activating mTOR, which was followed by hyperphosphorylation of TSC2, AMPK and acetyl CoA carboxylase (ACCα) [79]. Moreover, retransfection of p53-depleted HCT116 cells with wt p53 was shown to suppress autophagy [79,93].

One of the p53 target genes the TP53-induced glycolysis and apoptosis regulator (TIGAR) has been shown to fulfill an anti-autophagic functions [94]. Originally TIGAR was found to decrease fructose-2,6-bisphosphate (Fru-2,6-P2) levels in cells leading to the inhibition of glycolysis [94,95] TIGAR may also influence autophagy decreasing ROS production through increasing NADPH generation [96,97].

The mechanism by which cytoplasmic p53 inhibits autophagy is not completely understood [98]. However, there are some indications that this inhibition might be due to degradation of p53 which is promoted by numerous stress conditions such as ER stress, nutrient starvation or rapamycin [98]. Thus, autophagy induced by these stressors is suppressed by p53 stabilization. In turn, autophagy suppresses p53 significantly, thereby promoting tumorigenesis [98]. Recent studies underline the existence of the reciprocal interchange between autophagy regulation and p53 proteins. In a mouse model of mammary cancer, ablation of Palb2, which is the main binding partner of BRCA2, regulates its association with chromatin and functions in homologous recombination, leading to tumor development. Autophagy impairment, caused by monoallelic loss of the autophagy gene *Beclin 1*, limits mammary tumorigenesis driven by Palb2 deletion in a *wt Trp53*, but not in p53 null tumors [99]. Thus, the effect of autophagy on mammary tumorigenesis in Palb2-associated tumors is strongly dependent on p53 status.

As mentioned above, autophagy can reduce oxidative stress through the removal of organelles generating ROS, such as mitochondria and peroxisomes. On the other hand, oxidative stress leads to p53 activation, and thus, mitigating oxidative stress autophagy may suppress p53’s activity [100]. Autophagy may also influence p53 activation modulating the DNA damage process by providing substrates for DNA repair. Autophagy is also reported to suppress p53 through inhibition of AMPK, a major energy homeostasis regulator activated during starvation. p53 can also be degraded by chaperone-mediated autophagy [101]. Interestingly, mutant forms of p53 were preferentially found to localize in the cytoplasm and when transfected into p53-depleted cells could suppress autophagy. Only several isoforms of mut p53 were found to localize in the nucleus, including mutp53-P151H and mutp53-R282W, and they were not able to suppress autophagy [68]. The mechanisms of the interplay between autophagy and mut p53 were extensively reviewed by Cordani et al. [42]. All these data provide the evidence that cytoplasmic p53 might act as an inhibitor of autophagy. Thus, reciprocal interplay between autophagy regulation and p53 in cancer exists. Depending on localization p53 may inhibit or stimulate autophagy. Thus, nuclear p53 activates the autophagic pathway promoting cell resistance to the stress conditions, whereas cytoplasmic p53 suppresses autophagy favoring cell death. In turn, autophagy may contribute to the degradation of p53. Understanding the mechanisms involved in autophagy–p53 functional interplay may have important therapeutic implications and contribute to the development of new treatment strategies.

## 4. Autophagy and p53 in Regulation of Metastasis

Metastasis is a multi-step process that promotes cancer cell migration to distant organ sites [102,103]. The metastatic cascade can be subdivided into different stages, including local invasion, intravasation, survival in the circulation, extravasation, survival at a second site and finally outgrowth at a second site [104,105]. Epithelial-to-mesenchymal transition (EMT) is a biological process that allows epithelial cells to temporally acquire mesenchymal features by undergoing profound changes on molecular and biochemical levels [106,107]. EMT is also the principal biological phenomenon of metastasis occurring during cancer development since it maintains the ability of cancer cells to move from the original localization in order to colonize adjacent or distant sites [108].

The complex interplay between autophagy and p53 during tumorigenesis has been discussed above. However, it remains unclear whether they interact during the metastatic process. At the same time, multiple studies have shown that p53 and autophagy regulate the same processes at different stages of metastasis development [104,109].

**Figure 1:**
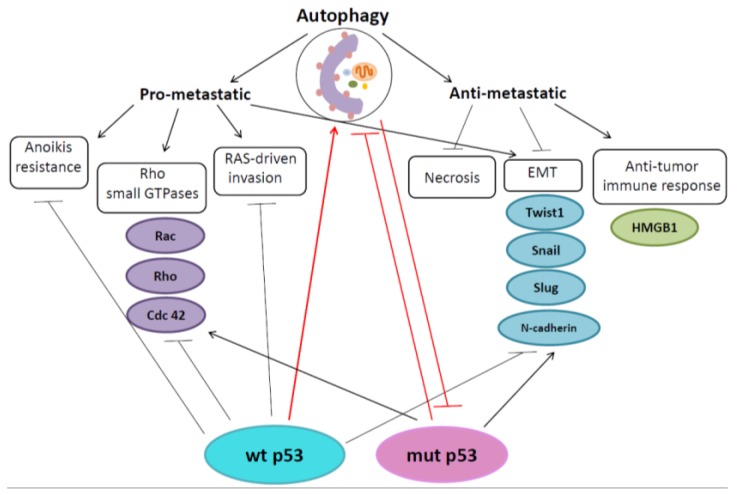
Schematic representation of interplay between p53, autophagy and metastasis. For details, see text. EMT- epithelial to mesenchymal transition, wt—wild type p53, mutp53—mutant p53.

In line with its dual role in cancer, the role of autophagy in the regulation of metastasis appears to be controversial and highly dependent on the cell type and/or tumor stage (Figure 1).

At the early stages of the metastatic process, autophagy may function as a suppressor of metastasis by reducing tumor necrosis, and inflammatory cell infiltration, and by mitigating oncogene-induced senescence [110]. At the same time, during later steps of the metastatic cascade, autophagy is supposed to promote this process stimulating detached cell survival and further colonization [107]. Interestingly, p53 was also shown to play a crucial role in the control of cell migration and invasion [109,111]. The wt p53 revealed the inhibition of metastasis regulating target genes involved in key metastasis pathways, including EMT, ECM interactions, cell migration and anoikis [112] whereas the introduction of mutant p53s correlates with a more invasive tumor phenotype, suggesting that mutant forms of p53 not only lose their tumor suppressor properties, but also gain pro-metastatic functions, which stimulate increased migration and motility of tumor cells (Figure 1) [113,114].

Specifically, autophagy may reduce inflammation, which is required for initiation of metastasis. Indeed, in promoting cell survival during various stress conditions, such as hypoxia, autophagy suppresses necrosis and further macrophage infiltration of the tumor site [47]. Autophagy can also contribute to inhibition of the metastatic process regulating the release of immunomodulatory factors HMGB1 from tumor cells (Figure 1) [115]. As mentioned above, once secreted, HMGB1 binds dendritic cell Toll-Like Receptor 4 on its surface, leading to an effective anti-tumor immune response that may eliminate tumor cells, preventing the spread of metastasis [116].

Autophagy has been shown to control EMT providing degradation of specific EMT regulators, such as Twist1, Snail and Slug, which are the transcription factors involved in the regulation of EMT [107,117,118]. Conversely, deficiency in autophagy leads to the upregulation and stabilization of Twist1, which, in turn, stimulates EMT activation promoting tumor growth and metastasis [119]. Interestingly, that stabilization of Twist1 in autophagy-defective cells is associated with the accumulation of SQSTM1/p62, which interacts with Twist1 and inhibits its degradation through either proteasomal or autophagosomal pathways (Figure 1). Moreover, EMT-inducing growth factors, such as TGFβ, favor an accumulation of SQSTM1/p62, which in turns stabilizes the SMAD4 and Twist1 promoting EMT [120]. Likewise, Grassi et al. have shown that autophagy promotes Snail degradation in an SQSTM1/p62-dependent manner, suppressing EMT and the migration of hepatocytes [121].

In turn, p53 may also act as a suppressor of metastasis, for example negatively regulating factors involved in the execution of the EMT program [92,122]. Overexpression of p53 in mammary epithelial cells that undergo EMT leads to their reversion back to an epithelial phenotype, a process known as mesenchymal–epithelial transition (MET) [123]. Similarly, activation of p53 in colorectal cells results in the acquisition of a more epithelial phenotype through MET. Since EMT is an essential step during metastatic progression, the ability of p53 to negatively regulate EMT may provide the explanation as to why p53 deficiency in different tumors correlates with a poor prognosis [124]. Conversely, p53 mutations are associated with high expression of EMT promoting Slug and low expression of E-cadherin, leading to poor prognosis of patients [28]. The study suggested that wt p53 can bind to MDM2 and Slug simultaneously to form a p53-MDM2-Slug complex, which then facilitates MDM2-mediated degradation of Slug [125].

As previously mentioned, autophagy may also promote metastasis regulating different stages of the metastatic cascade. Induction of autophagy sustains cancer cell survival under stress, which subsequently may promote tumor progression and metastasis [126,127]. Consistently with this idea, inhibition of autophagy by the deletion of FIP200, a ULK-interacting protein that is required for autophagosome formation, results in inhibition of metastasis mediated by an accumulation of damaged mitochondria and an increased level of ROS [128]. Likewise, inhibition of autophagy by bafilomycin A as well as *ATG7* knockdown reduced the expression of the mesenchymal markers vimentin and N-cadherin, thereby blocking EMT and subsequently migration of NSCLC cells [129].

Interestingly, autophagy also promotes resistance to anoikis, another form of programmed cell death occurring when cells detach from the ECM [130]. Autophagy activation during anoikis may be an important survival strategy allowing cells to overcome the stress caused by ECM detachment. Indeed, Fung et al. have reported that autophagy induced in epithelial cell lines protects cells from anoikis while ATG genes depletion results in apoptosis induction and reduced viability during the dissemination of tumor cells [131]. In breast cancer stimulation of autophagy in ECM-detached cells is associated with the generation of ROS and activation of the PERK pathway, which is ER-stress responsive kinase [132]. It is worth noting that suppression of p53 function leads to anoikis inhibition in thyroid epithelial cells, and detached transformed fibroblasts undergo anoikis only if they express wt p53 [133,134]. The capability of p53 to trigger anoikis could be associated with its role in the suppression of metastasis.

Autophagy may promote metastasis regulating the Rho family of small GTPases. Members of this family, including Rac, cdc42 and Rho, are small proteins that play a crucial role in the reorganization of actin cytoskeleton, and regulate the cell cycle progression, motility and invasion [135,136,137]. It has been established that the balance between the activities of Rac and Rho influences the way in which tumor cells migrate [138,139]. When Rac prevails, migration occurs with the elongated-morphology characteristic of tumor cells that have acquired a mesenchymal phenotype (Figure 1).

A recent study suggests that Rho A level is controlled by autophagy [140]. In contrast, inhibition of autophagy through ATG5 knockdown leads to aberrant accumulation of Rho A followed by the defects, cytokinesis multinucleation and aneuploidy required for cancer progression [140]. On the other hand, the Rho signaling pathway is involved in the regulation of autophagy. Thus, Rock1 knockdown enhanced autophagic flux characterized by the accumulation of enlarged autophagosomes. These data suggest that Rock signaling is involved in the regulation of the size of autophagosome by delimiting the phagophore elongation phase of early autophagy [141].

Additionally, it was demonstrated that the loss of p53 is associated with high level of GTP-bound Rho A, Rock1 and cdc42 [109]. In order to promote contractility and the ameboid mode of migration RhoA acts through its major downstream regulator Rock1, whereas the elongated mode is favored by Rac [138]. Thus, it was detected that the morphological changes occurring in p53−/− cells can result from an activation of cdc42 and/or RhoA/Rock signaling [142,143]. Moreover, mutant p53s regulate an ameboid migratory mode used by cancer cells in order to invade surrounding stroma which is promoted by increased RhoA/ Rock signaling [109].

Autophagy is also essential for RAS-driven oncogenic transformation and migration. Epithelial cells bearing RAS mutation display highly invasive phenotype associated with an EMT [144,145]. It was demonstrated that inhibition of autophagy in HRAS V12 MCF10A cells limits the formation of invasive protrusions and suppresses ECM proteolysis. In addition, autophagy inhibition reduces cell motility leading to reduced metastatic potential. However, the invasion capability of autophagy-defective HRAS V12 cells was restored, after treatment with conditioned media produced from autophagy-competent HRAS V12 cells, suggesting that autophagy promotes the production of growth factors driving migration and invasion in tumor cells [144]. On the other hand, several studies have shown that the loss of p53 can enhance RAS signaling-induced EMT [146] p53 may act as a checkpoint controller to inhibit EMT while the loss of p53 allows other signal cascades such as RAS activation to induce EMT [147,148]. Activation of K-RASV12 and the loss of p53 may cooperate to induce EMT and cell motility by triggering RhoA activity [146].

Taken together, these observations provide evidence that p53 and autophagy are involved in the regulation of different processes during metastatic progression including EMT and resistance to anoikis. However, despite close interaction during tumorigenesis the data about their direct interplay during metastasization are not clear enough. Better understanding of p53 perturbations in human cancer and their relationship with autophagy may be essential in order to provide more precise prognoses and improve therapeutic strategies.

## 5. Targeting p53 and Autophagy

Mutations of p53 are found in more than 50% of cancers. They are considered to be necessary for the development of most tumors. Their presence often contributes to rapid disease progression and resistance to conventional chemotherapy and is associated with poor prognosis, especially when the tumor bears gain-of-function mutant p53 [149,150]. Restoration of p53 function leads to regression of the tumor, especially in later stages of cancer [112,151,152,153]. Hence, significant efforts are made by researchers and clinicians to target p53 for treatment of a variety of tumor types.

The interplay between autophagy and mutant p53 is multifaceted. On the one hand, p53 has been demonstrated to inhibit autophagy, but on the other, chaperone-mediated autophagy triggers mutant p53 degradation and, thus, may regulate the progression of the tumor and its response to chemotherapeutic agents [93]. One can speculate, that impaired interplay between autophagy and p53, as well as consequent defects in the p53-dependent autophagy pathway may contribute to cancer progression. That means that restoration of wt p53—a novel and rapidly developing field in anticancer drug development—also normalizes autophagy. Several compounds, such as PRIMA-1, APR-246, a derivative and structural analogue of PRIMA-1; sulfonylpyrimidines such as PK11007; pyrazoles such as PK7088; zinc metallochaperone-1(ZMC1), a third-generation thiosemicarbazone as well as specific peptides have recently been shown to convert mutant p53 into a form exhibiting wt properties and thus to reactivate it [154,155,156] COTI-2 is a thiosemicarbazone-related compound, which not only targets p53, but also inhibits the PI3K/Akt/mTOR pathway thereby activating autophagy. Some of these compounds have been tested in preclinical models expressing mutant p53 and were proven to exhibit anticancer activity [157]. To date, two of these compounds, i.e. APR-246 and COTI-2 have progressed to clinical trials and are evaluated in patients with various gynecological cancers. Moreover, attempts have also been made to introduce a vector expressing p53. Gendicine (SiBiono, Shenzhen, China) is currently in clinical trial for various cancer types. It remains to be seen, however, whether any mutant p53 reactivating compound has the efficacy to combat metastasis [158].

Another approach to altering mutant p53 for therapy was suggested to target downstream pathways through which p53 acts. Despite the diversity of various p53 mutants, many of them share a common feature—they interact and inhibit p73, another potent tumor suppressor, which structurally and functionally resembles p53. A molecule called RETRA was shown to inhibit p73 and p53 interaction, thereby releasing p73 and activating transcription of its target genes, which suppress tumor growth [159]. One may also speculate, that RETRA could potentially impair the interaction of p53 with other proteins, but such data has not been provided yet.

Many types of cancer have been reported to evade cell death via inhibiting p53-dependent autophagy. It was found, for instance, that in renal cell carcinoma, transglutaminase 2 (TG2) crosslinks p53 in autophagosomes, thereby decreasing the p53 level in the cell and evading apoptosis [160]. Hence, there have also been attempts to restore autophagy and subsequent apoptosis in different cancer types via the p53-dependent pathway using different substances. It was found, for example, that cobalt chloride and β-asarone enhance the expression of p53, LC3-II/I, Beclin-1, AMPK and pAMPK and inhibit the expression of p62, Bcl-2, mTOR and pmTOR. They reduce the cell proliferation and promote p53-dependent autophagy in U251 cells and induce autophagy of human glioma cells and subsequent apoptosis [161,162]. In some recent studies it was demonstrated that tumor cells may be sensitized to radio- or chemotherapy by genetic or pharmacological intervention aimed at inhibiting autophagy. A series of data demonstrated that miR-148a-3p was downregulated in cisplatin-resistant gastric carcinoma cell lines and its reconstitution sensitized cells to cisplatin treatment through promoting mitochondrial fission and decreasing the AKAP1 expression level, which played a novel role in cisplatin resistance by inhibiting p53-mediated DRP1 dephosphorylation. MiR-148a-3p (microRNA-148a-3p) reconstitution in resistant cells inhibits cytoprotective autophagy by suppressing RAB12 expression and mTOR1 activation [163].

It is now clear that modulation of autophagy is promising therapeutic approach for certain cancer types [164,165]. However, although many autophagy-modulating drugs exist, some of which such as hydroxychlorochine are already employed in different clinical trials, many challenges remain [164]. Data about some of the recently introduced compounds, targeting autophagy, are summarized in Table 1.

Although in recent years there has been extensive discussion about whether inhibition of autophagy enhances or reduces cancer therapy, nowadays consensus has been reached that both may take place [176]. Rosenfeldt et al. demonstrated that p53 status may serve as a determinant of how autophagy influences the progression of pancreatic cancer. It was demonstrated that the loss of autophagy could be sufficient to avoid the progression of early-stage precancerous lesions into an advanced cancer. Moreover, it was shown that deficient autophagy in normal pancreatic tissue leads to elevation of p53 and consequent cell death [76].

Likewise, it was reported that mutant p53 becomes more stable when the cells are treated with the antimalarial drug hydroxychlorochine [177]. Another study has demonstrated that glucose restriction leads to autophagy-dependent degradation of mutant p53 protein. It was demonstrated that mutant p53 physically associates with several proteins presented in the autophagosomes [178]. Ultimately, mutant p53 degrades through macroautophagy, which, as mentioned above, is the pathway primarily used to eliminate misfolded proteins or damaged cellular organelles. Overall, stimulation of autophagy in cells with mutant p53 may promote cell death and depletion of the p53 level, thereby alleviating its oncogenic effects.

To sum up, the role of autophagy and its interplay with p53 depends on different factors and the clinical approach depends on the type of the tumor, p53 status and many other aspects. Future studies are required to confirm the efficacy of autophagy inducers in animal models and in cancer patients carrying the mutant *TP53* gene.

## 6. Concluding Remarks

There is ample evidence to indicate that a functional interplay between the tumor suppressor p53, its mutants and autophagy during tumorigenesis exists. An increasing number of studies also highlight the emerging roles of autophagy and p53 in the regulation of different metastatic cascade steps. Both p53 and autophagy were shown to contribute to anoikis resistance, EMT activation/suppression, ECM interactions, cell migration regulation, Ras-driven invasion, and several more. Importantly, at present, many issues remain unanswered. For example, how do p53 and autophagy interact biochemically during the metastatic process? How might the type of p53 mutation influence the relationship between autophagy and metastasis?

It is clear that the contribution of each of the different p53 target genes might vary according to the tumor type, tissue specificity, molecular context, stress signal and other circumstances. The contribution of autophagy in carcinogenesis is also context-dependent and today the consequences of its modulation are difficult to predict. Further studies are required in order to improve our understanding of the p53-autophagy-metastasis link and provide a new promising therapeutic approach to combat cancer.

## Figures and Tables

**Table 1:** Examples of compounds that restore autophagy and target p53-autophagy link.

Substance	Mechanism	Cancer Type	Reference
β-asarone	p53/Bcl-2/Bclin-1 and p53/AMPK/mTOR pathways	Glioma cell line (U251)	[161]
Walsuronoid B	ROS-formation and activation of p53/PI3K/Akt/mTOR signaling pathway	Liver cancer (HepG2 and Bel-7402)	[166]
Sulforaphane	Reduction of phosphorylation of Akt and mTOR	Malignant mesothelioma (H-28)	[167]
Physapubescin B	(ROS)-mediated suppression of mTORC1	Colon cancer (HCT116) and cervical cancer (HeLa)	[168]
Fluvastatin	p53/AMPK/mTOR pathway	Lung adenocarcinoma (A549 and SPC-A-1)	[169]
Trichostatin A and Valproic Acid	ROS formation	Pancreatic cancer (Panc1 and PaCa44)	[170]
Cobalt chloride	p53/Bcl-2/Beclin-1 pathway	Malignant glioma (U87-MG)	[162]
Oridonin	AMPK deactivation-mediated GLUT1 downregulation in p53-mutated cells	p53-mutated colorectal cancer cells (HCT-15, COLO205, HCT116, RKO, SW480, and SW620)	[171]
Astemizole-Histamine	ROS formation and p53 phosphorylation, which increased p53-p62 interactions to enhance Beclin-1-independent autophagy	breast cancer (MCF-7)	[172]
Polygonatum odoratum lectin	Up-regulation of miR-15a-3p, which mediated ROS-p53 pathway	human lung adenocarcinoma (A549)	[173]
Trichosanthin	ROS-formation and activation of p53/PI3K/Akt/mTOR signaling pathway,	gastric cancer (MKN-45)	[174]
Honokiol	ROS-formation and activation of p53/PI3K/Akt/mTOR signaling pathway	Glioma (U87 MG)	[175]
